# Association between Elevated Serum Tau Protein Level and Sepsis-Associated Encephalopathy in Patients with Severe Sepsis

**DOI:** 10.1155/2019/1876174

**Published:** 2019-07-17

**Authors:** Teng Zhao, Yan Xia, Dawei Wang, Li Pang

**Affiliations:** ^1^Department of Neurology, The First Hospital of Jilin University, Changchun 130021, China; ^2^Department of Gastroenterology, The First Hospital of Jilin University, Changchun 130021, China; ^3^Department of Emergency, The First Hospital of Jilin University, Changchun 130021, China

## Abstract

Sepsis-associated encephalopathy (SAE) is a common complication of sepsis. It is imperative to recognize, diagnose, and effectively manage SAE at the early stages. The aim of this study was to evaluate the potential of using the serum tau protein level in the diagnosis of SAE and the prediction of SAE outcomes. This was a retrospective and observational study. The patients included in this study were diagnosed with severe sepsis or septic shock. The serum tau protein level was measured using a commercial enzyme-linked immunosorbent assay. The association between the level of serum tau protein and SAE was assessed by multiple logistic regression analysis. One hundred nine patients with severe sepsis were enrolled during a period of two years. Of the 109 enrolled patients, 27 developed SAE. The serum tau protein level was significantly higher in the patients with SAE than that of the non-SAE group. The serum tau protein level and the sequential organ failure assessment (SOFA) score were independent factors that were associated with SAE. The combined use of the serum tau protein level with the SOFA score improved the accuracy in distinguishing SAE from non-SAE patients. A cutoff value serum tau protein level of 75.92 pg/mL had 81.1% sensitivity and 86.1% specificity in predicting the 28-day mortality in patients with severe sepsis. We identified a close association between the serum tau protein level with the appearance of SAE in patients with severe sepsis. The serum tau protein level can be useful in the prediction of poor outcomes in patients with sepsis.

## 1. Introduction

Sepsis is a severe syndrome that can involve the dysfunction and failure of multiple organs, including acute brain dysfunction. Sepsis-associated encephalopathy (SAE) is a common complication of sepsis that is characterized by altered mental activity, reduced attention, disorientation, delirium, or coma [1]. Although SAE is reversible, if no permanent brain damage occurs, survivors may suffer from permanent or irreversible cognitive impairment, leading to behavioral alteration and deteriorated life quality or early death [2]. Thus, it is imperative to recognize, diagnose, and effectively manage SAE at the early stages. However, there is a notable lack of specific and sensitive biomarkers for the diagnosis of SAE. Thus, SAE is underdiagnosed [3]. Because SAE pathophysiology remains poorly understood [4], exclusion methodology is used as a major clinical SAE diagnosis algorithm, in combination with case history, the state of consciousness, alterations in cognitive function, the Glasgow Coma Scale (GCS) score, neurological imaging examination, electroencephalography (EEG), and pathological findings. The nature of many of these symptoms is not objective and is often affected by the intervention of sedation and intubation. Furthermore, several of the diagnostic assays that are utilized in the diagnosis of SAE are invasive and have restricted clinical application. In addition, there are no reliable biomarkers for the prognosis of sepsis, particularly during the early stages of the disease. Multiple studies have attempted to identify such biomarkers in the field. For instance, the clinical value of several biomarkers in the prediction of SAE outcomes has been evaluated, including serum S100*β* [5], neuron-specific enolase (NSE) [6], vascular cell adhesion molecule-1 (VCAM-1) [7], calcium-binding protein A8 (S100A8), and tumor necrosis factor receptor-associated factor 6 (TRAF6) [8]. However, no consensus has been reached on the value of such biomarkers in the diagnosis of SAE.

Tau protein is a soluble microtubule-associated protein that is expressed by neurons and localized in the cytoplasm as well as in axons [9]. Tau protein may facilitate the assembly of tubulin molecules into microtubules, stabilizing microtubule structure and supporting the functioning of neurons. In addition, tau protein consists of axonal cytoskeleton and serves as an important traffic infrastructure, enabling the flow of proteins between the axonal terminus and the neuronal cell body. Tau protein can be released into the extracellular compartment, for instance, upon hypoxic or traumatic axonal injuries, and subjected to proteolytical cleavage. The cleaved product diffuses into the cerebrospinal fluid and blood. Tau protein has been studied as a biomarker for brain injury, and the potential for its use in the characterization of brain trauma, cerebral stroke, hypoxic ischemic encephalopathy, intracranial hemorrhage, and neurodegenerative disease clinically has been investigated [10–14]. However, no research has been conducted to characterize serum tau protein level and to explore the clinical application of this measurement in patients with sepsis. This study aimed to investigate whether there was a correlation between the serum tau protein level and the occurrence/outcomes of SAE in patients with sepsis.

## 2. Materials and Methods

### 2.1. Patients

This retrospective and observational study was conducted at the First Hospital of Jilin University. The patients included in this study were diagnosed with severe sepsis or septic shock in the emergency intensive care unit (ICU) between January 2016 and December 2017. The study protocol was approved by the Ethics Committee of the First Hospital, Jilin University. Written informed consent was obtained from each of the patients or a legally authorized relative.

Sepsis was diagnosed following the criteria that were established by the international guidelines for management of severe sepsis and septic shock [15]. Patients were excluded from the study if they had a previous diagnosis of a neuropsychiatric disease (head trauma, cerebral stroke, epilepsy, and intracranial infection), current brain disorders (hepatic encephalopathy, pulmonary encephalopathy, and severe electrolyte imbalance), concurrent hematologic diseases, malignant tumor, postcardiac arrest, or melanoma or if they were undergoing cancer chemotherapy.

### 2.2. Data Collection

Demographic, clinical, and laboratory data were retrieved after intensive care unit (ICU) admission from the medical records made by two physicians in emergency medicine. Age, gender, Acute Physiology and Chronic Health Evaluation II score (APACHE II score), Sequential Organ Failure Assessment (SOFA) score, GCS score, and infection sites were collected and determined during the first 24 hours of admission. The basic laboratory tests, including blood lactate, B-type natriuretic peptide, and inflammatory markers of white blood cell count (WBC), procalcitonin (PCT), and C-reactive protein (CRP), were detected on admission.

The GCS and mental status of the patients were evaluated twice a day, at eight o'clock in the morning and six o'clock in the afternoon, including SAE symptoms of somnolence, stupor, coma, confusion, disorientation, agitation, irritability, and decreased level of GCS. Sepsis-associated encephalopathy (SAE) was defined as cerebral dysfunction in the presence of severe sepsis as well as the presentation of two or more of the symptoms listed above after complete withdrawal of sedation. Original cerebral dysfunction derived from hypoxic encephalopathy, severe hypoglycemia, intracranial hemorrhage, epilepsy relapse, acute ischemic stroke, and hyponatremia were excluded. Supportive treatments, such as the use of a ventilator, length of ICU stay, and 28-day mortality, were also included for assessment.

### 2.3. Blood Sampling and Assays

Blood samples were collected from patients on admission through venipuncture. The resultant serum samples were aliquoted and stored at −80°C until further analysis. The serum tau protein level was measured using a commercial enzyme-linked immunosorbent assay (ELISA, BioSource hTAU Ag, Camarillo, CA). A standard curve-based formula was used to calculate the tau protein concentration of the tested samples. The lower detection limit of this kit was 15 pg/mL.

### 2.4. Statistical Analysis

Variable data are presented as mean ± SD (standard deviation) or median (quartile range). The significance of differences was assessed by the chi-square test for qualitative data, Student's *t*-test for normally distributed quantitative data, or Mann–Whitney *U* test for nonnormally distributed quantitative data. Multiple logistical regression analysis was used to identify the independent factors for the prediction of SAE outcomes using the forward stepwise method with the likelihood ratio test. Correlations between variables were tested by the Pearson linear regression test. Receiver operating characteristics (ROC) analysis was used to qualify marker performance, and ROC curves were constructed to assess the sensitivity, specificity, and respective areas under the curves (AUCs) of the tau protein performance with 95% CI. A value of *P* < 0.05 was considered statistically significant (SPSS 17.0 software package, SPSS, Inc., Chicago, IL, USA).

## 3. Results

### 3.1. Baseline Characteristics of the Included Patients

A total of 118 patients with severe sepsis were initially screened. Nine patients were excluded due to different etiologies for the altered consciousness, three had cardiopulmonary resuscitation related hypoxic encephalopathy, two with severe hyponatremia, and four with acute ischemic stroke. Among the 109 patients that met the inclusion criteria, 27 were diagnosed with SAE while 82 were diagnosed with non-SAE. The median hospital stay for SAE appearance was 2.5 day (0–6 days). The baseline characteristics of the patients are presented in Table 1.

The APACHE II and SOFA scores were significantly higher in the SAE group than in the non-SAE group (21.8 ± 6.7 versus 15.2 ± 4.6, *P*=0.013; 8.7 ± 2.6 versus 4.8 ± 3.3, *P* < 0.001, respectively). Among the laboratory tests, blood lactate was the only marker that was significantly higher in the SAE patients than in the non-SAE patients (3.86 ± 2.12 versus 2.40 ± 1.71, *P*=0.011). There were no significant differences in the infection source and culture results between the SAE and non-SAE groups.

Averaged mechanical ventilation was significantly higher in the SAE group (8.6 ± 10.8 days) than in non-SAE group (3.1 ± 5.2 days; *P*=0.017). The average ICU stay duration in the SAE group was significantly higher than in the non-SAE group (11.5 ± 16.2 vs. 7.7 ± 8.2 days; *P*=0.023). The 28-day mortality rate was significantly higher in the SAE group (51.9%, 14/27) than in the non-SAE group (26.8%, 22/82; *P*=0.016).

### 3.2. Serum Tau Protein Level and Sepsis Severity

**Figure 1:**
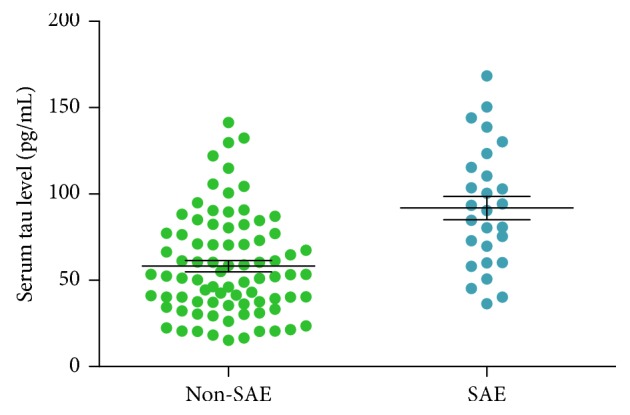
The range of serum level tau protein in each group. The average serum level of tau protein is higher in the SAE group than that of the non-SAE group (91.90 ± 35.14 pg/mL vs. 58.18 ± 29.17 pg/mL; *P* < 0.001). The black horizontal lines in each group indicate the mean level and standard error ranges.

The average serum tau protein level in the SAE group was significantly higher than that of the non-SAE group (91.90 ± 35.14 vs. 58.18 ± 29.17 pg/mL; *P* < 0.001). The ranges of serum tau protein level between the two groups are presented in Figure 1. Pearson's correlation analysis showed that the serum tau protein level correlated with the SOFA score (*r* = 0.769, *P*=0.001) and the APACHE II score (*r* = 0.664, *P*=0.008).

### 3.3. Factors Associated with the Outcome of SAE

**Figure 2:**
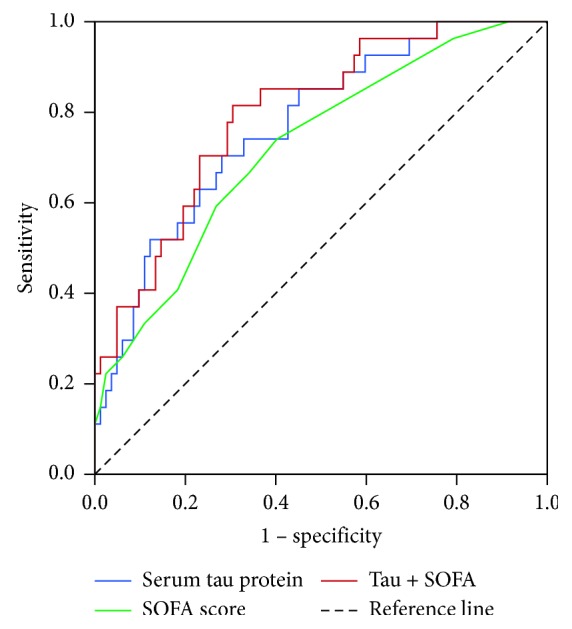
Receiver operating characteristic (ROC) curve for the sensitivity and specificity of serum tau protein level, SOFA score, or both in predicting SAE among patients with severe sepsis. All of the parameters shown above were calculated to obtain an optimal predicted probability for the serum tau protein level and SOFA score that were set at 71.96 pg/mL and 6, respectively. The optimal predicted probabilities of the serum tau protein level, SOFA score, and a combination of the two were obtained from their respective ROC curves by maximizing the sum of the sensitivity and specificity and minimizing the overall error (square root of the sum [1 − sensitivity]^2^ + [1 − specificity]^2^) and by minimizing the distance of the cutoff value to the top-left corner of the ROC curve.

The only independent factors that were identified as being associated with SAE occurrence and outcomes through multiple logistic regression analysis were the serum tau protein level (odds ratio (OR), 1.537; 95% CI, 1.136–1.945, *P*=0.001) and the SOFA score (OR, 0.664, 95% CI 0.308–0.912, *P*=0.002). The ROC curve determined that a serum tau protein level >71.96 pg/mL (AUC: 0.770, 95% confidence interval (CI): 0.671–0.869) can predict SAE with 70.4% sensitivity and 72.0% specificity. An optimal SOFA score cutoff value was set at 6 (AUC: 0.723, 95% CI: 0.615–0.832, sensitivity: 66.7%, specificity: 65.9%). The predictive values and likelihood ratios for the serum tau level and SOFA score in SAE prediction are displayed in Table 2. The serum tau protein level displayed greater AUC, sensitivity, and specificity values than the SOFA score in distinguishing SAE cases from non-SAE cases (Figure 2, Table 2). All performances were enhanced when the serum tau protein level and the SOFA score were combined for diagnosis and prediction of SAE (Figure 2, Table 2).

### 3.4. Impact of Serum Tau Protein Levels on 28-Day Mortality

**Figure 3:**
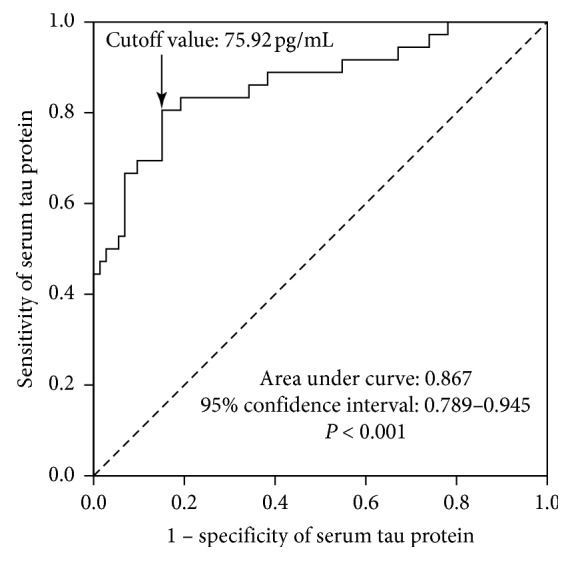
The performance of the serum tau protein level-based analysis of the 28-day mortality among patients with severe sepsis, as evaluated by receiver operating characteristic (ROC). The optimal cutoff value of serum tau protein level of 75.92 pg/mL predicted the 28-day mortality with 81.1% sensitivity and 86.1%, specificity (AUC: 0.867, 95% CI: 0.789–0.945).

The mortality rate within 28 days among the 109 patients with severe sepsis was 33% (36/109). The average serum tau protein level was significantly higher among the patients that did not survive than in the patients that survived (97.09 ± 33.42 pg/mL vs. 51.46 ± 21.99 pg/mL; *P* < 0.001). The ROC determined that a serum tau protein level >75.92 pg/mL predicted the 28-day mortality among patients with severe sepsis, which displayed 81.1% sensitivity and 86.1% specificity (AUC: 0.867, 95% CI: 0.789–0.945; Figure 3).

## 4. Discussion

A reported 9%∼71% of patients with sepsis may experience encephalopathy, an indicator for poor outcomes, including a 16% to 63% hospital mortality rate [16]. In our study, 24.8% of the patients with severe sepsis developed SAE, and 51.9% of the SAE patients died. Our study showed that the serum tau protein level was an independent indicator for SAE occurrence, and it also served as a predictor for 28-day survival among patients with severe sepsis.

Several factors, including age, SOFA, APACHE II and GCS score, lactic acidosis, and other parameters, are associated with SAE development and death [7, 17]. However, the performance of these factors in different clinical settings has not been highly reproducible. In the present study, a higher SOFA score was the only indicator for the development of SAE in patients with severe sepsis. The SOFA score is known as a nonspecific indicator for the severity of various diseases and has also been used to evaluate the severity and outcome in SAE [17]. Thus, our finding was expected and valid.

Tau protein is a small phosphorylated protein that mainly appears in the axonal compartment of neurons [18]. Serum tau protein is mainly derived from injured axons that release tau protein into the extracellular space. Thus, an elevated serum tau protein level is likely to reflect brain injury, as indicated by investigations into brain injury biomarkers. A sustained elevation of the tau protein level may indicate persistent neurological damage [12, 19]. To enter the blood, tau protein must pass through the blood-brain barrier (BBB). Thus, SAE pathogenesis may cause increased BBB permeability and/or disruption.

This study represents the first investigation of both the diagnostic and prognostic value of the detection of the serum tau protein level in patients with SAE. As shown by stepwise logistic regression and AUC analysis, the serum tau protein level was more accurate in predicting SAE among patients with severe sepsis than SOFA score. ROC analysis set a serum tau protein level of 71.96 pg/mL as the optimal cutoff value for the development of SAE. This value displayed a predictive value of 70.4% sensitivity and 72.0% specificity, which was a better predictive value than that of the SOFA score (66.7% sensitivity and 65.9% specificity). The combination of the serum tau level with the SOFA score led to further improvements in the performance. Our findings indicate that serum tau protein levels may serve as an additional marker that can be used to assess the severity of sepsis and the development of SAE. This finding may improve SAE management.

As indicated by several previous studies, the serum tau protein level is useful in corroborating the finding of acute neuronal injury, including acute ischemic stroke, traumatic brain injury, intracranial hemorrhage, epilepsy, and cardiac arrest [11–13, 19, 20]. In the current study, we correlated the serum tau level with the 28-day clinical outcome and found that the serum tau protein level was significantly higher in the group that did not survive 28 days than in the group that did survive 28 days. ROC curve analysis set a tau protein level of >75.92 pg/mL as the cutoff for predicting the 28-day mortality. This value achieved 81.1% sensitivity, 86.1% specificity, and an AUC value of 0.867 in this cohort. Thus, another function of the serum tau protein level is to predict the outcome of patients with SAE.

It is widely accepted that a good marker should show ≥80% of specificity and sensitivity. This was achieved by tau for the prediction of 28-day mortality of SAE. For the diagnosis of SAE, the specificity and sensitivity of tau and SOFA only reached 65.9% and 72.0%, respectively. The combination of the serum tau protein level and SOFA achieved >80% sensitivity; however, the specificity remained at a suboptimal level (70.0%). Thus, although the use of the measurement of serum tau protein level is promising, this measurement fell short in both sensitivity and specificity for the diagnosis of SAE in this cohort.

There are a few limitations of this study. First, the sample size was small, and thus there was not enough statistical power in the analyses. Second, kinetic serum tau levels were not investigated because no additional serum samples were available for testing. The detected differences in the serum tau protein level may include kinetic variations among different patients. Nevertheless, our study presents the preliminary evidence that the serum tau level may function as an additional marker to assist in the early diagnosis of SAE and the accurate prediction of the outcome of SAE.

## Figures and Tables

**Table 1:** Baseline demographic and clinical findings between the SAE and non-SAE groups.

Items	Total (*n* = 109)	SAE (*n* = 27)	Non-SAE (*n* = 82)	*P* value
Age, *y*, mean ± SD	61.7 ± 13.0	64.4 ± 14.2	57.8 ± 12.5	0.142

Male/Female	64/45	16/11	48/34	0.386

Disease severity, mean ± SD				
APACHE II score	18.4 ± 5.2	21.8 ± 6.7	15.2 ± 4.6	0.013^*∗*^
SOFA score	6.3 ± 3.1	8.7 ± 2.6	4.8 ± 3.3	<0.001^*∗*^

Inflammatory markers				
WBC, mean ± SD (×10^9^/L)	15.24 ± 9.90	16.54 ± 9.67	13.81 ± 10.17	0.373
PCT, mean ± SD (ng/mL)	35.24 ± 27.81	30.77 ± 26.32	38.24 ± 30.46	0.214
CRP, mean ± SD (mg/L)	193.84 ± 91.21	196.92 ± 87.84	188.47 ± 96.45	0.307

Biochemistry				
Lactate, mean ± SD (mmol/L)	3.12 ± 2.03	3.86 ± 2.12	2.40 ± 1.71	0.011^*∗*^
Glucose, mean ± SD (mmol/L)	8.95 ± 3.07	9.35 ± 3.32	8.71 ± 2.69	0.413
Creatinine, mean ± SD (*μ*mol/L)	135.74 ± 28.61	142.46 ± 31.40	126.45 ± 26.91	0.092
BUN, mean ± SD (mmol/L)	12.27 ± 9.76	13.08 ± 10.26	11.44 ± 9.34	0.194
Albumin, mean ± SD (g/L)	25.06 ± 5.86	24.75 ± 6.28	26.35 ± 5.32	0.316
Total bilirubin, mean ± SD (mmol/L)	22.31 ± 5.82	24.52 ± 6.33	21.41 ± 5.06	0.481
AST, median (range) (U/L)	346 (43–12016)	395 (48–12016)	320 (43–9830)	0.106
ALT, median (range) (U/L)	156 (25–8900)	172 (25–8900)	142 (34–7500)	0.085
Cholesterol, mean ± SD (mmol/L)	3.44 ± 0.47	2.78 ± 0.42	4.01 ± 0.50	0.126
BNP, median (range) (pg/mL)	1710 (520–12800)	1740 (570–12800)	1630 (520–9200)	0.124
pH, mean ± SD	7.40 ± 0.11	7.39 ± 0.09	7.40 ± 0.13	0.437
PaO_2_, mean ± SD (mmHg)	77.26 ± 43.21	78.32 ± 44.85	76.37 ± 42.09	0.753
PaCO_2_, mean ± SD (mmHg)	43.02 ± 19.43	42.65 ± 18.44	44.69 ± 20.79	0.321
HCO_3_^−^, mean ± SD (mmol/L)	24.01 ± 4.05	25.62 ± 4.21	23.18 ± 3.50	0.416

Infection sites				0.473
Respiratory system	39 (35.8)	9 (33.3)	30 (36.6)	
Urinary system	32 (29.4)	6 (22.3)	26 (31.7)	
Blood	17 (15.6)	5 (18.5)	12 (14.6)	
Digestive system	12 (11.0)	4 (14.8)	8 (9.8)	
Skin or soft tissue	6 (5.5)	2 (7.4)	4 (4.9)	
Other	3 (2.7)	1 (3.7)	2 (2.4)	

Bacteriological categories				0.228
Gram-negative bacteria	70 (64.2)	16 (59.3)	54 (65.9)	
Gram-positive bacteria	35 (32.1)	9 (33.3)	26 (31.7)	
Fungi	4 (3.7)	2 (7.4)	2 (2.4)	
Mechanical ventilation days	6.5 ± 7.6	8.6 ± 10.8	3.1 ± 5.2	0.017^*∗*^
Days in the ICU	9.3 ± 12.7	11.5 ± 16.2	7.7 ± 8.2	0.023^*∗*^
28-day mortality	33.0%	51.9%	26.8%	0.016^*∗*^

APACHE: Acute Physiology and Chronic Health Evaluation; SOFA: Sequential Organ Failure Assessment; WBC: white blood cells; PCT: procalcitonin; CRP: C-reactive protein; BUN: blood urea nitrogen; AST: aspartate aminotransferase; ALT: alanine aminotransferase; BNP: B-type natriuretic peptide. ^*∗*^*P* < 0.05.

**Table 2:** Relative performance of serum tau protein, SOFA score, or a combination of both^*∗*^ in predicting SAE in patients with severe sepsis.

	AUC (95% CI)	Sensitivity (%)	Specificity (%)	PPV (%)	NPV (%)	Positive LR	Negative LR
Tau protein	0.770 (0.671–0.89)	70.4	72.0	45.3	88.1	2.514	0.411
SOFA score	0.723 (0.615–0.82)	66.7	65.9	39.2	85.7	1.956	0.505
Tau protein + SOFA score	0.798 (0.705–0.890)	81.5	70.0	47.3	92.0	2.717	0.264

AUC, area under the curve; PPV, positive predictive value; NPV, negative predictive value; LR, likelihood ratio; CI, confidence interval; SOFA: Sequential Organ Failure Assessment. ^*∗*^The diagnostic cutoff values for serum tau protein and SOFA score were 71.96 pg/mL and 6, respectively.

## Data Availability

The data used to support the findings of the present study are included within the article.
